# Disinfection of the Hands

**Published:** 1891-04

**Authors:** 


### Disinfection of the Hands.

Franz Boll (*Deutsche med. Wochenschrift*, No. 17, 1890), in the course of some experiments upon the best means of disinfecting the hands of the surgeon, in which the methods employed by the bacteriologists were used to demonstrate freedom from germs by gelatin cultures, comes to the conclusion that the course pursued in Mikulicz's clinic in Konigsberg offers the best known practical means of accomplishing this object. This consist of vigorously brushing the hands for not less than three minutes with potash soap and water, after which they are immersed for half a minute each, first in a three per cent. solution of carbolic acid, and then in a 1-2000 sublimate solution. Finally, the subungual spaces and fold are thoroughly rubbed and cleansed with ten per cent. iodoform gauze which has been dipped in a five per cent. solution of carbolic acid. Experiments made to determine the efficiency of simple soap and water cleansing, showed this to be entirely unreliable.—*Brooklyn Med. Journal.*

